# High-Selectivity Proton Exchange Membranes with Low Ion Exchange Capacity and Hydrophobic Side Chain-Induced Micro-Phase Separation for Vanadium Redox Flow Batteries

**DOI:** 10.3390/membranes16050170

**Published:** 2026-05-06

**Authors:** Li Tian, Huixiang Yao, Bo Pang, Wanting Chen, Fujun Cui, Qining Wang, Yujie Guo, Xuemei Wu, Xiaobin Jiang, Gaohong He

**Affiliations:** 1 State Key Laboratory of Fine Chemicals, Research and Development Center of Membrane Science and Technology, School of Chemical Engineering, Dalian University of Technology, Dalian 116024, China; 2 Panjin Institute of Industrial Technology, Dalian University of Technology, Panjin 124221, China; 3 School of Chemical Engineering, Ocean and Life Sciences, Dalian University of Technology, Panjin 124221, China

**Keywords:** hydrophobic side chains, low ion exchange capacity, proton-conductive membrane, H^+^/V^n+^ selectivity, vanadium redox flow batteries

## Abstract

The proton (H^+^) and vanadium ion (V^n+^) selectivity of proton-conductive membrane is one of the key components for vanadium redox flow batteries (VRFBs). In this work, a hydrophobic side chain was designed to accelerate proton conduction with high selectivity of H^+^ and V^n+^ for the VRFB membrane. The grafting of hydrophobic butyl side chains into the membrane (PBIOSO_3_-But) induced the formation of a high microphase separation capacity to form large and connected ion conductive channels with low ion exchange capacity (IEC). As a result, the PBIOSO_3_-But membrane with low IEC of 1.26 mmol g^−1^ shows area resistance of 0.19 Ω cm^2^ as well as vanadium permeability of 3.2 × 10^−9^ cm^2^ s^−1^, leading to a high H^+^/V^n+^ selectivity of 2.51 × 10^10^ mS s cm^−3^ (higher than Nafion 212, 4.62 × 10^8^ mS s cm^−3^). Notwithstanding its low ion-exchange capacity, this membrane demonstrates H^+^/V^n+^ selectivity surpassing that of recently reported microphase separation membranes. Compared to the Nafion 212 membrane (74.3% EE; 0.81% per cycle), the PBIOSO_3_-But membrane exhibited superior VRFB performance, achieving an energy efficiency of 83.2% at 200 mA cm^−2^ and a low retention rate of 0.22% per cycle. These values compare favorably with those of recently reported membranes.

## 1. Introduction

**Figure 1:**
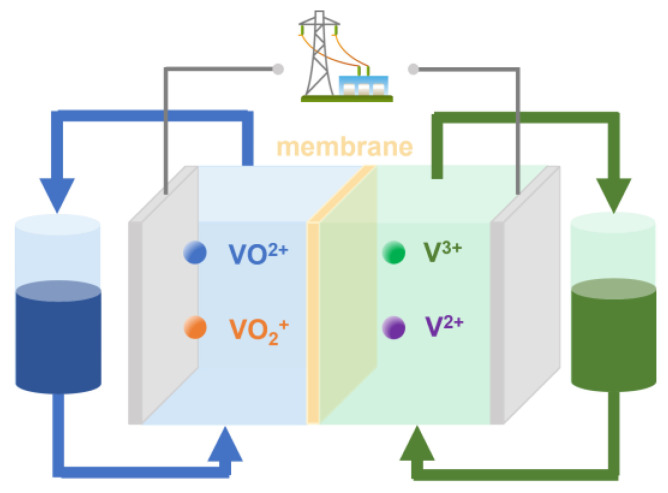
The schematic diagram of the VRFB system.

Vanadium redox flow batteries (VRFBs) have become a research focus in the field of large-scale energy storage technology due to their prominent advantages in design flexibility, operational safety, and life cycle. They are particularly suitable for addressing the inherent intermittency of renewable energy generation, such as wind and solar power, while ensuring grid stability and power quality [1,2,3]. In VRFBs, the energy can be stored and released by shuttling between the different oxidation states of vanadium ions. Featuring a scalable design, inherent safety, and decoupling of capacity and power, this technology effectively meets the requirements for integrating, storing, and regulating renewable energy [4,5,6]. A schematic diagram of the VRFB system is shown in Figure 1. The proton-conductive membrane is one of the crucial elements in VRFBs [7,8]. It enables high battery performance by simultaneously blocking the crossover of vanadium ions (V^n+^) and conducting protons (H^+^) [9,10]. However, this trade-off effect between low V^n+^ permeability and high H^+^ conductivity remains a fundamental bottleneck to simultaneously achieving high proton conductivity and selectivity in proton-conductive membranes [11,12]. Therefore, developing proton-conductive membranes that simultaneously achieve high proton conductivity and high H^+^/V^n+^ selectivity to reduce ohmic polarization remains a key challenge for VRFBs.

To achieve high proton conductivity and high H^+^/V^n+^ selectivity, a well-defined microphase-separated structure, composed of hydrophobic and hydrophilic domains, can be constructed within the membrane through rational side-chain chemical design. This architecture facilitates the formation of a hydrogen-bonding network. The interconnected hydrophilic domains provide rapid, low-resistance pathways for H^+^, the charge carriers, thereby mitigating ohmic polarization caused by impeded ionic conduction. Concurrently, the dense matrix of the hydrophobic domains serves as an effective support, enhancing the mechanical stability of the membrane [13,14,15,16]. Conventionally, hydrophilic domains can be designed via different ion conductive groups design in the side chain (-SO_3_H [15], -COOH [17], -OSO3H [18], etc.) to accelerate proton conduction with high H^+^/V^n+^ selectivity. Furthermore, by designing the hydrophobic domains through the chemical structure design of hydrophobic side chains, the microphase separation formation ability can be synergistically enhanced with the hydrophilic ion-conductive groups to construct efficient conductive pathways, thereby improving both proton conductivity and H^+^/V^n+^ selectivity [15,19,20,21,22]. For instance, Hu et al. [20] incorporated fluoroalkyl side chains with varying carbon numbers onto a poly (aryl piperidinium) backbone to construct interconnected hydrophilic channels for anion transport. As the fluorine content in the polymer increased, its hydrophilicity decreased significantly, while the anion transport capacity first increased and then decreased. Lindström et al. [21] modified poly (p-terphenyl) with fluorine-containing groups, synthesizing two polymers pendant with perfluoroalkyl and perfluorophenyl groups, respectively. Among these, the PEM containing perfluorophenyl groups exhibited a higher degree of microphase separation enhancement and a higher ion exchange capacity, leading to superior ionic conductivity. Unlike the conventional approach of direct graft modification onto polymer backbones, Li et al. [19] adopted a distinct strategy to enhance the performance of Nafion membranes by incorporating fluoroalkyl side chain-grafted polyoxometalates as supramolecular additives, which were precisely anchored within the ionic clusters of Nafion. Specifically, within the hydrophilic regions formed by the sulfonic acid groups of Nafion, the introduction of hydrophobic fluorine moieties significantly amplified the microphase separation between the hydrophilic and hydrophobic domains, promoting the formation of more defined and interconnected ionic clusters. This morphological optimization facilitated more efficient proton hopping and transport through the membrane, ultimately yielding a 56% enhancement in proton conductivity for the composite membrane compared to pristine Nafion. However, the introduction of hydrophobic side chains leads to discrete ion clusters and increases vanadium permeability, which consequently limits the enhancement of proton conductivity and H^+^/V^n+^ selectivity.

**Scheme 1:**
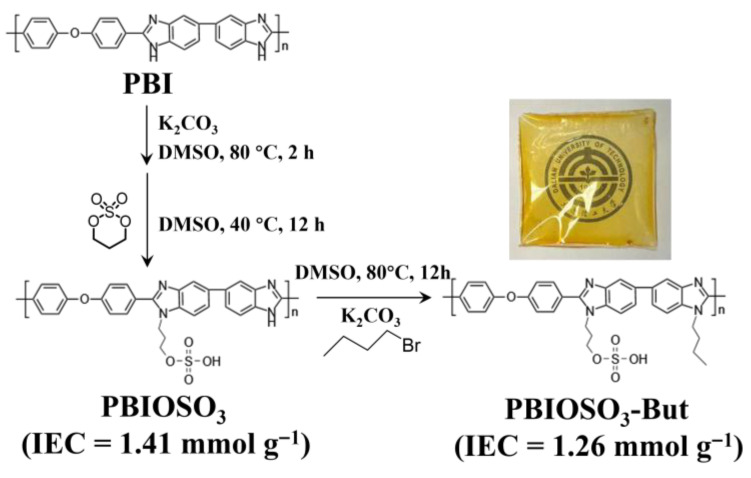
Preparation of **butyl group**-grafted PBIOSO_3_ [18] and PBIOSO_3_-But.

In this work, a hydrophobic butyl side chain was designed to enhance microphase separation capacity in the sulfated ion-conductive membrane with low ion exchange capacity (IEC, Scheme 1). After the introduction of the hydrophobic butyl side chain (-But), the ion cluster size increases from 7.14 nm to 7.85 nm. However, the IEC decreased from 1.41 mmol g^−1^ to 1.26 mmol g^−1^. The PBIOSO_3_-But membrane combines low area resistance of 0.19 Ω cm^2^ and low vanadium permeability of 3.2 × 10^−9^ cm^2^ s^−1^ due to its enlarged ion clusters and decreased IEC. As a result, it exhibits an exceptional selectivity of 2.51 × 10^10^ mS s cm^−3^, surpassing that of Nafion 212 (4.62 × 10^8^ mS s cm^−3^). Moreover, the VRFB performance of the PBIOSO_3_-But membrane presents 83.2% energy efficiency at 200 mA cm^−2^ and retention of 0.22% per cycle, better than Nafion 212 membrane (74.3% EE at 200 mA cm^−2^ and retention of 0.79% per cycle). These values compare favorably with those of recently reported microphase-separated membranes.

## 2. Materials and Experimental Methods

### 2.1. Materials

The materials and chemical reagents employed in this study were as follows. Polybenzimidazoles (PBI) were supplied from Shengjun Plastic (Shanghai, China). Anhydrous potassium carbonate (K_2_CO_3_), 1,3-propanediol cyclic sulfate, and 1-bromobutane were procured from Aladdin Scientific (Shanghai, China). The Nafion 212 membrane was obtained from DuPont (Wilmington, DE, USA). The vanadium solution used as the VRFB electrolyte was prepared from vanadium oxide sulfate hydrate, purchased from Aladdin Scientific (Shanghai, China). All organic solvents, including dimethyl sulfoxide (DMSO), anhydrous ethanol, and ethyl acetate, were purchased from Tianjin Damao Chemical Reagent Factory (Tianjin, China). All commercially available chemicals were of analytical grade and were used as received without any further purification.

### 2.2. Synthesis of Sulfate-Grafted PBI (PBIOSO_3_) and Butyl-Grafted PBIOSO_3_(PBIOSO_3_-But)

The synthesis of the sulfate-grafted PBI (PBIOSO_3_) followed our previous report [18], and the synthesis of butyl-grafted PBIOSO_3_ (PBIOSO_3_-But) was based on PBIOSO_3_. To prepare a solution, a 4 g portion of PBI was weighed and dissolved in DMSO (200 mL) (Scheme 1). An amount of 10.6 g of K_2_CO_3_ was added to the solution, and the mixture was stirred at 80 °C for 4 h. After the system was naturally cooled to 40 °C, 10.6 g of 1,3-propanediol cyclic sulfate was added under Ar, and the synthetic reaction was continued with stirring at this temperature for 12 h. After the reaction, the crude product was washed with ethyl acetate, ethanol, and deionized water, followed by collection as the solid target product, designated PBIOSO_3_. The product was dried under vacuum and then dissolved in DMSO to prepare the casting solution.

The synthesis of the butyl-grafted PBIOSO_3_ (PBIOSO_3_-But) requires the prior preparation of a PBIOSO_3_ casting solution. An amount of 100 mL of PBIOSO_3_ casting solution was placed under Ar, followed by the addition of 2.7 g of anhydrous potassium carbonate and 177 µL of 1-bromobutane. The solution was then stirred at 80 °C for 12 h. The same washing procedure was applied, giving the final PBIOSO_3_-But casting solution.

After being cast onto a clean glass plate, the PBIOSO_3_-But solution was subjected to thermal drying in an oven maintained at 80 °C for 24 h to ensure complete solvent evaporation. The resulting membranes were transparent and homogeneous, with a thickness measured to be approximately 20 ± 2 μm using a digital micrometer, confirming successful film formation and uniform thickness distribution.

### 2.3. Structural and Physical Characterization

Nuclear magnetic resonance spectroscopy (NMR; AVANCE NEO 600M, Karlsruhe, Germany) and Fourier transform infrared spectroscopy (FTIR; Thermo Fisher iS50, Waltham, MA, USA) confirmed the structure of the products. ^1^H NMR analysis allowed quantification of the grafted sulfate groups (the degree of substitution, DS), from which the IEC was calculated using the following equation. (${\bar{M}}_{{PBIOSO}_{3}}$ and ${\bar{M}}_{PBI}$ represents the molar mass of the repeating unit of PBIOSO_3_ and PBI).

(1) $$IEC=\frac{DS}{{\bar{M}}_{{PBIOSO}_{3}}\times DS+{\bar{M}}_{PBI}\times \left(1-DS\right)}$$

The surface and cross-section of the membrane were observed by scanning electron microscopy (SEM; JSM 6300F, Tokyo, Japan), while the ion clusters were examined by transmission electron microscopy (TEM; JEM-F200, Tokyo, Japan) and small-angle X-ray scattering (SAXS; Xeuss 3.0, Grenoble, France). Prior to TEM imaging, the membrane was immersed in a 0.5 M AgNO_3_ solution at 25 °C for 24 h to enable the ion cluster imaging. A universal testing machine (Shimadzu; AGS-X, Kyoto, Japan) was employed to evaluate the mechanical properties of the membranes.

The water uptake of the membrane was determined through immersion experiments at room temperature [10]. The cast proton exchange membrane was first dried under vacuum at 60 °C for one day, and then immersed in deionized water at room temperature with stirring for one day. After removal, the membrane was gently wiped to remove surface moisture, and its thickness and mass were subsequently measured. The water uptake was calculated as the ratio of the mass difference before and after immersion to the mass of the dried membrane; the swelling ratio was determined as the ratio of the increase in diameter to the diameter of the dried membrane.

To evaluate the vanadium ion permeability (P), a diffusion cell was employed. One compartment was charged with 1.5 M VOSO_4_ in 3 M H_2_SO_4_, while the other contained 1.5 M MgSO_4_ in 3 M H_2_SO_4_ [11]. At 12 h intervals, the concentration of VO^2+^ in the MgSO_4_ solution was determined using a UV-Vis spectrophotometer (Shimadzu; UV-2600i, Kyoto, Japan). P was calculated using Equation (2). It is worth noting that the conversion between concentration and absorbance in 762 nm is carried out through a standard curve.

(2) $$V_{B}\frac{d\left(C_{B}\left(t\right)\right)}{dt}=A\frac{P}{L}\left(C_{A}-C_{B}\left(t\right)\right)$$

To evaluate the ex situ chemical stability, a membrane sample (2 cm × 2 cm) was immersed in 30 mL of 1.5 M VO_2_^+^ solution (in 3 M H_2_SO_4_) and kept at 25 °C for 30 days. During this period, every 10 days, the VO^2+^ concentration was determined using UV-Vis spectrophotometry at a wavelength of 762 nm.

Electrochemical impedance spectroscopy (EIS) was employed to determine the area resistance (AR) of the membrane [12]. Measurements were carried out at 25 °C in a diffusion cell containing 3 M H_2_SO_4_, using a Technologies electrochemical workstation (Ivium; Eindhoven, The Netherlands). AR was derived from Equation (3). Here, R_1_ and R_2_ represent the impedance with and without the membrane, respectively.

(3) $$AR=\left(R_{1}-R_{2}\right)S$$

The in-plane proton conductivity of the membrane was measured by EIS using a four-electrode system at room temperature. The measurement frequency ranged from 10 Hz to 10^7^ Hz with an amplitude of 10 mV [12]. The direct current in-plane proton conductivity was determined from the Nyquist plot of the real part of the complex conductivity as a function of alternating current frequency.

The mechanical properties of the membranes were evaluated using a universal testing machine (AGS-X; Shimadzu, Kyoto, Japan). Prior to testing, the membrane samples were cut into a standardized rectangular shape with uniform width (1 cm) and were clamped to the testing grips at the same marked length to ensure proper alignment and avoid pre-stressing. During the test, the crosshead speed was set to 5 mm min^−1^. Stress–strain curves were obtained at room temperature, from which the tensile strength and elongation at break of the membranes were derived.

The performance of the VRFBs was tested by LANHE battery system (CT2001A; Wuhan, China). The membrane was pretreated by soaking in 1 M H_2_SO_4_ for 24 h before use. A single cell was then tested at 25 °C. Additionally, 1.5 M, 20 mL V^2+^ and V^3+^ in 3 M H_2_SO_4_ was used as the negative electrolyte, while 1.5 M, 20 mL VO^2+^ and VO_2_^+^ in the same H_2_SO_4_ served as the positive electrolyte. At a constant current density of 200 mA cm^−2^, the cell was subjected to 100 charge–discharge cycles within a voltage range of 0.8–1.65 V to evaluate its stability. The coulombic efficiency (CE) and voltage efficiency (VE) were determined using Equations (4) and (5). It is worth noting that the energy efficiency (EE) used to evaluate the overall performance of the battery is determined by Equation (6), which is the product of CE and VE. Here, I/V stands for current/voltage, and the subscripts c/d represent charging and discharging.

(4) $$CE=\frac{\int I_{d}dt}{\int I_{c}dt}\times 100\%$$

(5) $$VE=\frac{\int V_{d}I_{d}dt}{\int V_{c}I_{c}dt}\times 100\%$$

(6) EE=VE×CE×100%

## 3. Results and Discussion

### 3.1. Chemical Structure of PBIOSO_3_-But Membrane

**Figure 2:**
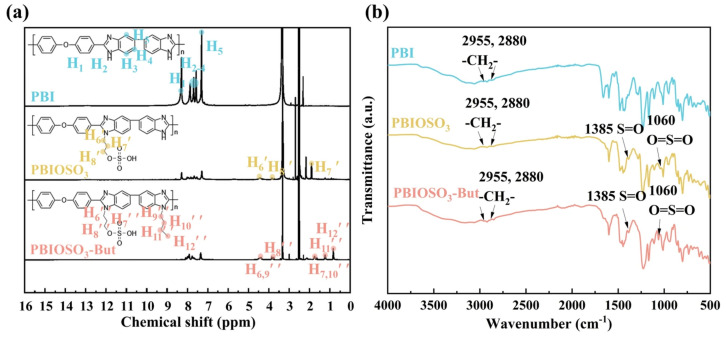
(**a**) ^1^H-NMR and (**b**) FTIR spectra of the PBI, PBIOSO_3_, and PBIOSO_3_-But membranes.

The ^1^H-NMR and FTIR were used to characterize the chemical structures of the PBI, PBIOSO_3_, and PBIOSO_3_-But membranes. As shown in Figure 2a, the new hydrogen signals H_6_′, H_8_′ and H_7_′ were arisen at 4.5, 3.9 and 1.9 ppm, which indicate the successful grafting of sulfate ester side chain in the membrane. After the grafting of hydrophobic butyl side chains, the characteristic peaks of H_9_″, H_10_″, H_11_″, H_12_″ appeared at 4.5, 1.9, 1.3 and 0.8 ppm. Thus, the degree of substitution (DS) for the sulfate ester side chains and the degree of grafting (GS) for the butyl side chains in the PBIOSO_3_-But membrane can be calculated by DS = 2 × S [H_8_″]/S [H_1_″] and GS = 2 × S [H_11_″]/S [H_1_″], respectively, where S [H_n_] represents the integrated peak area of the corresponding hydrogen (Figure S1). Therefore, the DS was 70%, and the GS was calculated to be 102%. According to Equation 1, the IEC of PBIOSO_3_ was calculated to be 1.41 mmol g^−1^ and the IEC of PBIOSO_3_-But was calculated to be 1.26 mmol g^−1^. The successful preparation of PBIOSO_3_-But was further validated by characteristic peaks observed in the FTIR spectrum (Figure 2b). Compared with PBI and PBIOSO_3_ membranes, the characteristic peaks at 1385 cm^−1^ (S=O) and 1060 cm^−1^ (O=S=O) can be detected, which confirm the successful grafting of sulfate ester side chains onto the membrane. Moreover, the -CH_2_- peaks at 2955 cm^−1^, 2880 cm^−1^ can also be confirmed which indicates the successful co-grafting of hydrophobic butyl side chains and sulfate ester side chains onto the PBI main chain.

### 3.2. Membrane Morphology of PBIOSO_3_-But Membrane

**Figure 3:**
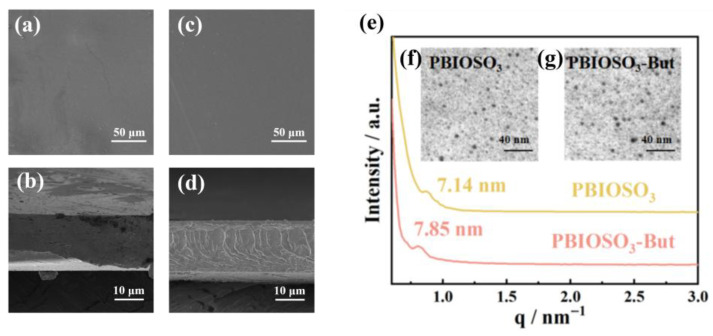
Morphology of PBIOSO_3_ and PBIOSO_3_-But. (**a**) PBIOSO_3_ membrane, SEM images, surface. (**b**) PBIOSO_3_ membrane, SEM images, cross-section. (**c**) PBIOSO_3_-But membrane, SEM images, surface. (**d**) PBIOSO_3_-But membrane, SEM images, cross-section. (**e**) SAXS of PBIOSO_3_ and PBIOSO_3_-But. (**f**,**g**) TEM images (**f**) PBIOSO_3_ and (**g**) PBIOSO_3_-But.

The surface and cross-sectional morphologies of the membranes were observed by SEM. As shown in Figure 3a–d, both the pristine PBIOSO_3_ membrane and PBIOSO_3_-But membrane exhibit smooth and homogeneous surfaces without any visible defects, pinholes, or cracks. The cross-sectional images further reveal a dense and uniform internal structure across the entire membrane thickness, confirming the formation of densely packed polymer matrices. To quantitatively investigate the nanoscale microphase-separated morphology, small-angle X-ray scattering (SAXS) analysis was conducted on the membranes. As shown in Figure 3e, both membranes exhibit characteristic scattering peaks corresponding to ionic aggregates. Notably, despite its lower IEC, the PBIOSO_3_-But membrane displays a scattering peak shifted to a smaller scattering vector (q) compared to the pristine PBIOSO_3_ membrane. According to Bragg’s law, the calculated ion cluster size increases from 7.14 nm for the PBIOSO_3_ membrane to 7.85 nm for the PBIOSO_3_-But membrane. This apparent contradiction—the formation of larger ion clusters despite a lower IEC—is attributed to the strategic introduction of hydrophobic butyl side chains. The presence of these hydrophobic moieties amplifies the thermodynamic incompatibility between the hydrophilic segments and the hydrophobic polymer backbone. This enhanced driving force for phase separation promotes more effective microphase separation, enabling the limited number of ionic groups to aggregate into larger and more well-defined ionic clusters. This microphase-separated morphology is further evidenced by TEM images (Figure 3f,g), where the dark spots represent hydrophilic ionic clusters and the bright regions correspond to the hydrophobic polymer matrix. The ion cluster sizes obtained from TEM are consistent with those from SAXS, and the size distribution is shown in Figure S2. Compared with the PBIOSO_3_ membrane, the PBIOSO_3_-But membrane displays larger ion cluster size, confirming its enhanced microphase separation capability upon the grafting of hydrophobic butyl side chains.

**Figure 4:**
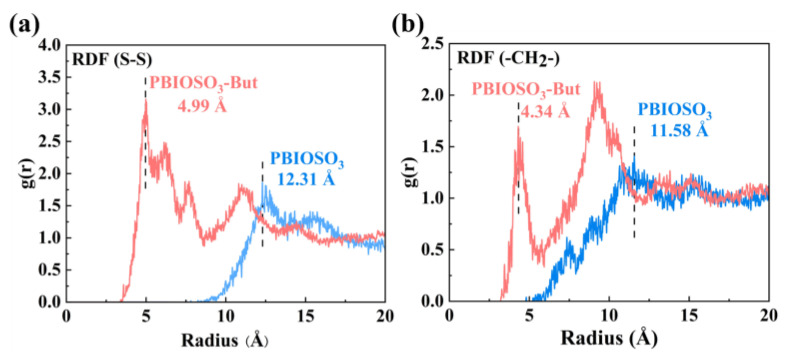
Radial distribution function (RDF) simulations of the PBIOSO_3_ and PBIOSO_3_-But membrane. (**a**) RDF between the S groups and (**b**) RDF between the -CH_2_- groups on hydrophilic segments.

The PBIOSO_3_-But membrane achieves high microphase separation capability through the grafting of hydrophobic butyl side chains. As shown by the radial distribution function (RDF) in Figure 4, the PBIOSO_3_-But membrane exhibits enhanced characteristic peak signals compared to the PBIOSO_3_ membrane. The first prominent peaks indicate that the distance between sulfur atoms on adjacent hydrophilic side chains (S-S, Figure 4a) decreases to 4.99 Å, while the distance between methylene (-CH_2_-, Figure 4b) groups on neighboring hydrophilic segments reduces to 4.34 Å. These RDF results confirm that the hydrophilic sulfate ester groups are arranged more closely and orderly in space, providing direct evidence for enhanced microphase separation and the formation of ion-conductive channels. This optimized microstructure enables the PBIOSO_3_-But membrane to maintain a low IEC while forming highly efficient ion-conductive channels.

### 3.3. Properties of the PBIOSO_3_-But Membrane

**Figure 5:**
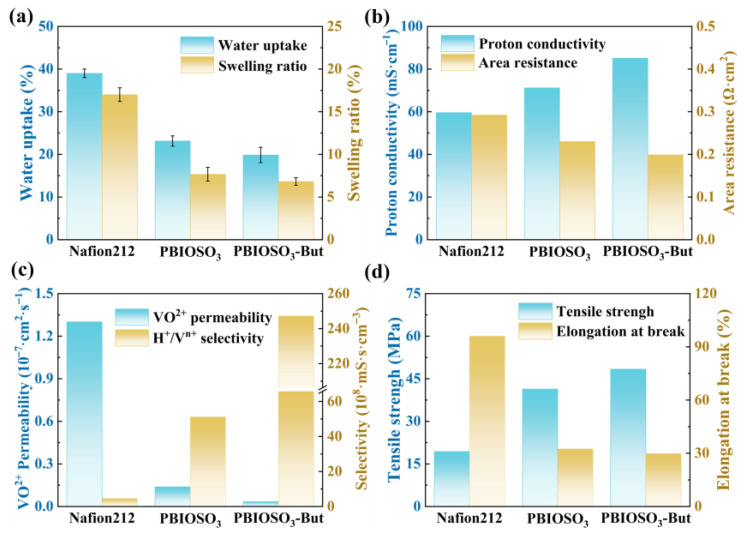
Fundamental properties of the Nafion 212, PBIOSO_3_, PBIOSO_3_-But membrane. (**a**) Swelling ratio and water uptake, (**b**) conductivity of proton and AR, (**c**) permeability of vanadium and H^+^/V^n+^ selectivity, (**d**) mechanical properties.

The incorporation of hydrophobic butyl side chains significantly altered the properties of the PBIOSO_3_ membrane, as shown in Figure 5. First, the water uptake and swelling ratio of the membranes were investigated to evaluate their dimensional stability (Figure 5a). Due to the decreased IEC resulting from butyl side chain grafting, the PBIOSO_3_-But membrane exhibited a reduced water uptake of 19.8% and a swelling ratio of 7%, demonstrating better dimensional stability compared to the unmodified PBIOSO_3_ membrane. This suppressed water absorption behavior is beneficial for maintaining the structural integrity of the membrane during operation.

In terms of in-plane proton conduction performance, despite its lower IEC, the PBIOSO_3_-But membrane achieved a high proton conductivity of 85.09 mS cm^−1^, outperforming both the Nafion 212 and PBIOSO_3_ membranes (Figure 5b). Correspondingly, its area resistance was as low as 0.19 Ω cm^2^, significantly lower than that of the reference samples. This result indicates that the enhanced microphase separation capability induced by the hydrophobic side chains effectively constructed interconnected hydrophilic ion transport channels, compensating for the adverse effects of reduced IEC and achieving improved proton conduction performance. Regarding vanadium ion barrier properties, the PBIOSO_3_-But membrane exhibited an extremely low V^n+^ permeability of only 3.2 × 10^−9^ cm^2^ s^−1^. This represents a 97.4% reduction compared to Nafion 212 (1.30 × 10^−7^ cm^2^ s^−1^) and a 75.2% reduction compared to the PBIOSO_3_ membrane (1.39 × 10^−8^ cm^2^ s^−1^). This excellent vanadium barrier performance is primarily attributed to two factors: first, the reduced IEC leads to decreased water uptake and swelling; second, the dense hydrophobic domains effectively block the crossover of vanadium ions. Based on the aforementioned excellent proton conduction and vanadium ion barrier properties, the PBIOSO_3_-But membrane achieved a H^+^/V^n+^ selectivity as high as 2.51 × 10^10^ mS s cm^−3^, which is 4.83 times that of the PBIOSO_3_ membrane and significantly superior to the Nafion 212 membrane (Figure 5c). In terms of mechanical properties, the introduction of hydrophobic side chains significantly reduced the water uptake and swelling ratio of the membrane, endowing the PBIOSO_3_-But membrane with excellent mechanical strength. Its tensile strength reached 47.4 MPa, much higher than that of Nafion 212 (19.3 MPa) and also superior to that of the PBIOSO_3_ membrane (41.4 MPa). Meanwhile, its elongation at break was 29.7%, lower than that of Nafion 212 (96.0%) and the PBIOSO_3_ membrane (32.5%). This indicates that the introduction of hydrophobic side chains enhances the interactions between polymer chains, forming a more rigid network structure that is beneficial for improving the durability of the membrane during long-term operation (Figure 5d).

**Figure 6:**
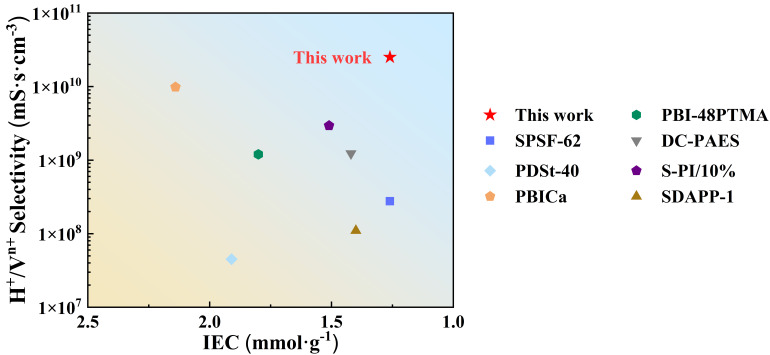
Comparison of IEC and H^+^/V^n+^ selectivity for recently reported microphase separation membranes [22,23,24,25,26,27,28].

Notwithstanding its relatively low IEC of only 1.26 mmol g^−1^, the PBIOSO_3_-But membrane demonstrates exceptional H^+^/V^n+^ selectivity that surpasses not only the pristine PBIOSO_3_ membrane and commercial Nafion 212 but also the majority of recently reported microphase-separated membranes in the literature (Figure 6) [22,23,24,25,26,27,28].

### 3.4. VRFB Performance Test

**Figure 7:**
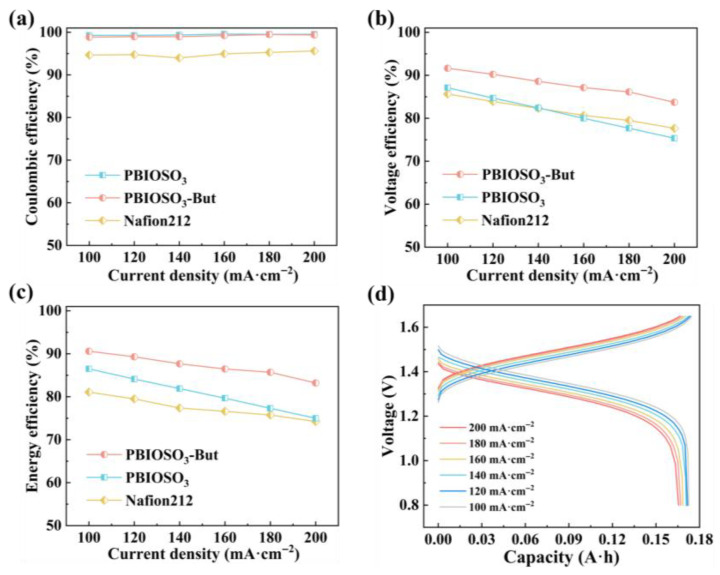
VRFB Performance of single cells with membranes. (**a**–**c**) Efficiency: (**a**) CE, (**b**) VE, (**c**) EE, (**d**) charge–discharge curves.

To validate the practical applicability of the as-prepared membranes, their VRFB performance was evaluated in a single-cell configuration. As shown in Figure 7, the coulombic efficiency (CE), voltage efficiency (VE), and energy efficiency (EE) of VRFB single cells assembled with Nafion 212, PBIOSO_3_, and PBIOSO_3_-But membranes were systematically compared over a current density range of 100 to 200 mA cm^−2^. Notably, both the pristine PBIOSO_3_ membrane and the PBIOSO_3_-But membrane exhibited coulombic efficiencies exceeding 99% across the entire current density range (100–200 mA cm^−2^), substantially higher than that of the Nafion 212 membrane (Figure 7a). As discussed previously, this superior CE performance is directly attributed to the exceptionally low vanadium ion permeability of the PBIOSO_3_-based membranes. The dense membrane structure effectively suppresses vanadium ion crossover, thereby minimizing self-discharge reactions during charge–discharge cycling. In particular, the incorporation of hydrophobic butyl side chains in the PBIOSO_3_-But membrane plays a crucial role in enhancing its voltage efficiency. The butyl side chains promote microphase separation, leading to the formation of well-connected hydrophilic domains that serve as efficient proton transport pathways. This optimized morphology facilitates rapid proton conduction and reduces ohmic polarization, resulting in the highest VE among all tested membranes (Figure 7b). The synergistic effect of high CE and enhanced VE enables the PBIOSO_3_-But membrane to achieve superior EE: 90.6% at 100 mA cm^−2^ and 83.2% at 200 mA cm^−2^ (Figure 7c). These EE values represent a significant improvement over both the unmodified PBIOSO_3_ membrane (86.5% at 100 mA cm^−2^ and 75.0% at 200 mA cm^−2^) and the Nafion 212 membrane (81.1% at 100 mA cm^−2^ and 74.3% at 200 mA cm^−2^). The charge–discharge curves of PBIOSO_3_-But at various current densities are shown in Figure 7d.

**Figure 8:**
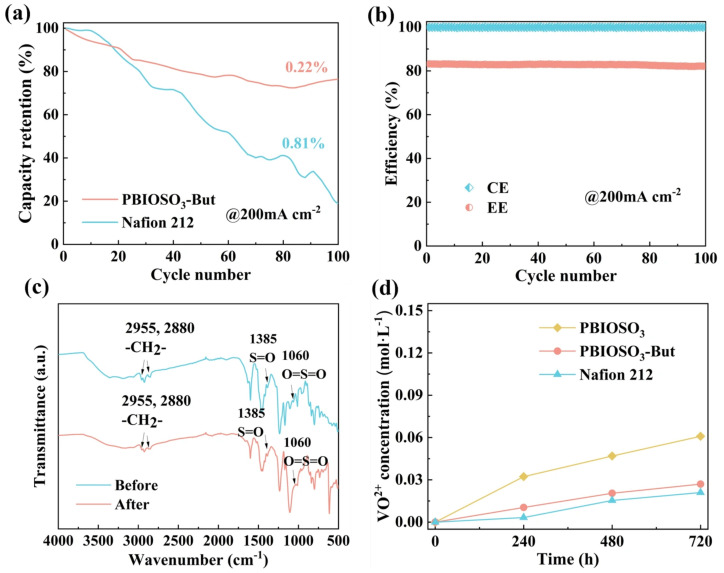
Chemical durability and cycling test of PBIOSO_3_-But. (**a**) Capacity retention of cycling test, (**b**) 100 cycles test at 200 mA cm^−2^, (**c**) FTIR spectra of PBIOSO_3_-But before and after the cycling test, (**d**) chemical stability test of membranes.

The PBIOSO_3_-But membrane demonstrated both a low capacity decay rate and high chemical stability. As shown in Figure 8a, after 100 charge–discharge cycles, the membrane retained 77.6% of its initial discharge capacity, corresponding to a minimal capacity decay rate of only 0.22% per cycle. This value is substantially lower than that of the Nafion 212 membrane, which exhibited a decay rate of 0.81% per cycle under identical testing conditions. The significantly suppressed capacity fade highlights the effectiveness of the PBIOSO_3_-But membrane in mitigating vanadium ion crossover and maintaining long-term cycling durability. Furthermore, even when subjected to a high current density of 200 mA cm^−2^, the PBIOSO_3_-But membrane maintained excellent cycling stability (Figure 8b). The chemical stability of the PBIOSO_3_-But membrane was systematically evaluated through post-cycling FTIR analysis and ex situ chemical stability tests. As shown in Figure 8c, the FTIR spectra of the PBIOSO_3_-But membrane after cycling were compared with those of the pristine membrane. The characteristic absorption bands corresponding to the sulfate ester groups—specifically the S=O stretching vibration at 1385 cm^−1^ and the O=S=O stretching vibration at 1060 cm^−1^—exhibited no obvious peak shift or intensity change. Similarly, the aliphatic C–H stretching vibrations originating from the grafted butyl side chains (-CH_2_- stretching at 2955 cm^−1^ and 2880 cm^−1^) remained virtually unchanged. This spectroscopic evidence confirms that the chemical structure of the PBIOSO_3_-But membrane, including both the hydrophilic sulfate ester groups and the hydrophobic butyl side chains, remains intact after prolonged electrochemical cycling, demonstrating its excellent structural stability under operating conditions. Furthermore, after immersion in a strongly oxidizing solution for 720 h, the PBIOSO_3_-But membrane exhibited superior oxidative stability compared to the pristine PBIOSO_3_ membrane, as evidenced by the lower VO^2+^ concentration. This enhanced resistance to oxidative degradation is attributed to the presence of the hydrophobic butyl side chains, which form a dense, protective hydrophobic matrix that effectively shields the polymer backbone from attack by highly oxidative vanadium ions.

## 4. Conclusions

In this study, we report a polybenzimidazole-based ion-conductive membrane grafted with hydrophobic side chains (PBIOSO_3_-But), which exhibits an enhanced microphase-separated structure and a low ion exchange capacity (IEC). Following the introduction of hydrophobic butyl side chains into the PBIOSO_3_ membrane (IEC of 1.41 mmol g^−1^), the incompatibility between the hydrophilic and hydrophobic components of the polymer is enhanced, leading to tighter aggregation of the hydrophilic domains and improved microphase separation. Consequently, even at a low IEC (1.26 mmol g^−1^), large-sized and continuously connected ion-conductive channels are constructed. After the introduction of the hydrophobic butyl side chain (-But), the ion cluster increases from 7.14 nm to 7.85 nm. This structural optimization results in a reduced area resistance (0.19 Ω cm^2^) and vanadium ion permeability (3.2 × 10^−9^ cm^2^ s^−1^), achieving an ultrahigh H^+^/V^n+^ selectivity of 2.51 × 10^10^ mS s cm^−3^. This value is 54.3 times higher than that of the commercial benchmark Nafion 212 membrane (4.62 × 10^8^ mS s cm^−3^). Both the H^+^/V^n+^ selectivity and IEC of this membrane surpass those of the majority of recently reported microphase-separated membranes. Remarkably, the PBIOSO_3_-But membrane demonstrates outstanding performance in vanadium redox flow batteries (VRFBs), achieving an energy efficiency (EE) of 83.2% at a current density of 200 mA cm^−2^ with a minimal decay rate of only 0.22% per cycle. This performance outperforms that of the Nafion 212 membrane (EE of 74.3% and a decay rate of 0.81% per cycle). This work provides critical insights into the rational chemical structural design of high-performance membrane materials for VRFB applications.

## Data Availability

The original contributions presented in this study are included in the article. Further inquiries can be directed to the corresponding authors.

## Supplementary Materials

The following supporting information can be downloaded at: https://www.mdpi.com/article/10.3390/membranes16050170/s1, Figure S1: The explanation of the meanings of the area ratios of each NMR peak, Figure S2: Size distribution of ion clusters in TEM images, Table S1: Through-plane swelling ratio of the PBIOSO_3_ and PBIOSO_3_-But membranes, Table S2: Mechanical properties of the PBIOSO_3_-But membrane under dry and wet conditions.
