# Supplementary material for: High-Selectivity Proton Exchange Membranes with Low Ion Exchange Capacity and Hydrophobic Side Chain-Induced Micro-Phase Separation for Vanadium Redox Flow Batteries
Source: Membranes (Basel). 2026 May 6;16(5):170. doi: 10.3390/membranes16050170 (PMC13208432; doi:10.3390/membranes16050170)
Supplement: Supplementary file 1 [file membranes-16-00170-s001.zip › membranes-4216668-supplementary.pdf]

*membranes*

*Supplementary Materials*

# High-Selectivity Proton Exchange Membranes with Low Ion Exchange Capacity and Hydrophobic Side Chain-Induced Micro-Phase Separation for Vanadium Redox Flow Batteries

**Li Tian [1], Huixiang Yao [1], Bo Pang [1,*], Wanting Chen [1], Fujun Cui [2], Qining Wang [1], Yujie Guo [1], Xuemei Wu [1,3], Xiaobin Jiang [1], Gaohong He [1,3,*]**

[1] State Key Laboratory of Fine Chemicals, Research and Development Center of Membrane Science and Technology, School of Chemical Engineering, Dalian University of Technology, Dalian 116024, China; tianli010430@mail.dlut.edu.cn (L.T.); 1912262082@mail.dlut.edu.cn (H.Y.); chenwanting@dlut.edu.cn (W.C.); 17836033867@mail.dlut.edu.cn (Q.W.); guoyj9@mail.dlut.edu.cn (Y.G.); xuemeiw@dlut.edu.cn (X.W.); xbjiang@dlut.edu.cn (X.J.)

[2] Panjin Institute of Industrial Technology, Dalian University of Technology, Panjin 124221, China; haozhecui@foxmail.com

[3] School of Chemical Engineering, Ocean and Life Sciences, Dalian University of Technology, Panjin 124221, China

* Correspondence: pangb@dlut.edu.cn (B.P.); hgaohong@dlut.edu.cn (G.H.)

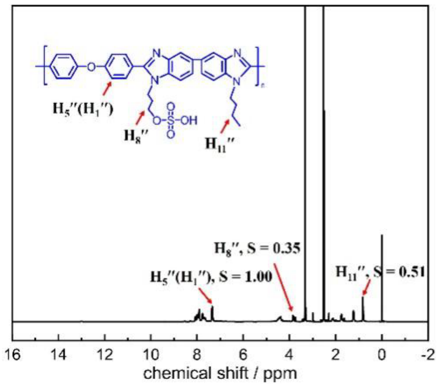


**Figure S1.** The explanation of the meanings of the area ratios of each NMR peak.

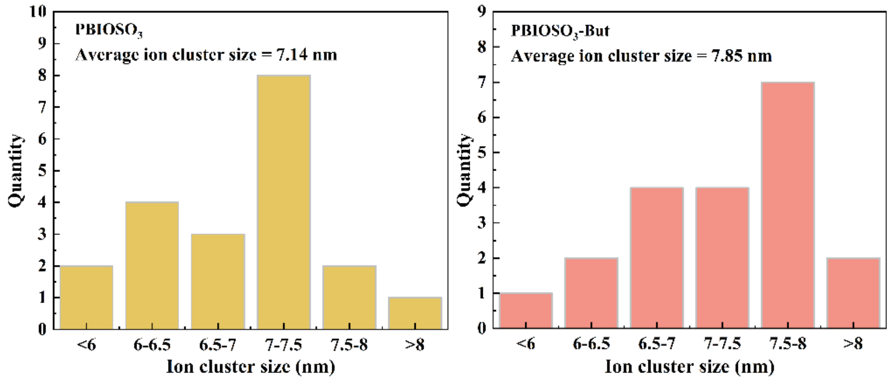


**Figure S2.** Size distribution of ion clusters in TEM images.

**Table S1.** Through-plane swelling ratio of the PBIOSO$_3$ and PBIOSO$_3$-But membranes.

| Through-plane swelling | Before [mm] | After [mm] | Swelling ratio [%] |
|---|---|---|---|

| | | | |
|---|---|---|---|
| $PBIOSO_3$ | 0.025 | 0.027 | 8.0% |
| $PBIOSO_3$-But | 0.024 | 0.026 | 4.2% |

**Table S2.** Mechanical properties of the $PBIOSO_3$-But membrane under dry and wet conditions.

| **$PBIOSO_3$-But** | **Tensile strength [%]** | **Elongation at break [%]** |
|---|---|---|
| dry | 48.39 | 29.83 |
| wet | 44.13 | 30.18 |
